# On synchronal algorithm for fixed point and variational inequality problems in hilbert spaces

**DOI:** 10.1186/s40064-016-1723-6

**Published:** 2016-02-01

**Authors:** L. M. Bulama, A. Kılıçman

**Affiliations:** Department of Mathematics, Universiti Putra Malaysia (UPM), 43400 Serdang, Selangor Malaysia

**Keywords:** Synchronal algorithm, Total strict asymptotically pseudocontraction, K-strict pseudo-contraction, Nonexpansive mapping, Fixed point and variational inequality problem, 47H09, 47H10

## Abstract

The aim of this article is to expand and generalize some approximation methods proposed by Tian and Di (J Fixed Point Appl, 2011. doi:10.1186/1687-1812-21) to the class of $(k, \{\mu _{n}\}, \{\xi _{n}\}, \phi )$-total asymptotically strict pseudocontraction to solve the fixed point problem as well as variational inequality problem in the frame work of Hilbert space. Further, the results presented in this paper extend, improve and also generalize several known results in the literature .

## Background

Let $\left\langle .,.\right\rangle$ be an inner product, $\left\| .\right\|$ be the corresponding norm and *H* be a Hilbert space. The mapping $T:H\rightarrow H$ is said to be; nonexpansive, if $\left\| Tx-Ty\right\| \le \left\| x-y\right\| , \forall x,y\in H$, quasi-nonexpansive, if $\left\| Tx-q\right\| \le \left\| x-q\right\| , \forall x\in H$ and $q\in Fix(T)$, $\eta$-strongly monotone, if there exists a positive constant $\eta >0$ such that $\left\langle Tx-Ty,x-y\right\rangle \ge \eta \left\| x-y\right\| ^{2}, \forall x,y\in H$, uniformly *L*-Lipschitzian, if there exists $L>0$ such that $\left\| T^{n}x-T^{n}y\right\| \le L\left\| x-y\right\|$, $\forall x,y\in H$ and *T* is said to be strongly positive bounded linear operator, if there is a constant $\gamma >0$ such that $\left\langle Tx,x\right\rangle \ge \gamma \left\| x\right\| ^{2}, \forall x\in H,$ and also *T* is said to be; contraction if there exists a constant $\beta \in [0,1)$ such that $\left\| Tx-Ty\right\| \le \beta \left\| x-y\right\| , \forall x,y\in H$, strictly pseudocontraction if there exists a constant $k\in [0,1)$ such that

$$\begin{aligned} \left\| Tx-Ty\right\| ^{2}\le \left\| x-y\right\| ^{2}+ k\left\| (I-T)x-(I-T)y\right\| ^{2}, \quad \forall x,y\in H. \end{aligned}$$

The mapping *T* is said to be; asymptotically strict pseudocontraction if there exists a constant $k\in [0,1)$ and a sequence $\{k_{n}\}\subset [1,\infty )$ with $k_{n}\rightarrow 1$ as $n\rightarrow \infty$ such that

$$\begin{aligned} \left\| T^{n}x-T^{n}y\right\| ^{2}\le k_{n}\left\| x-y\right\| ^{2}+ k\left\| (I-T^{n})x-(I-T^{n})y\right\| ^{2}, \quad \forall n\ge 1 ~\mathrm{and}~ x,y\in H, \end{aligned}$$

$$(k, \{\mu _{n}\}, \{\xi _{n}\}, \phi )$$-total asymptotically strict pseudocontraction, if there exists a constant $$k\in [0,1)$$, $$\mu _{n}\subset [0,\infty )$$, $$\xi _{n}\subset [0,\infty )$$ with $$\mu _{n}\rightarrow 0$$ and $$\xi _{n}\rightarrow 0$$ as $$n\rightarrow \infty$$, and continuous strictly increasing function $$\phi :[0,\infty )\rightarrow [0,\infty )$$ with $$\phi (0)=0$$ such that $$\left\| T^{n}x-T^{n}y\right\| ^{2}\le \left\| x-y\right\| ^{2}+ k\left\| (I-T^{n})x-(I-T^{n})y\right\| ^{2} +\mu _{n}\phi (\left\| x-y\right\| ) +\xi _{n},$$$$\forall x,y\in H.$$

We now give an example of $(k,\; \{\mu _{n}\}, \{\xi _{n}\}, \phi )$-total asymptotically strict pseudocontraction mappings .

### *Example 1*

Let *B* be a unit ball in a real Hilbert space $l_{2}$ and $T:B\rightarrow B$ be a mapping define by

$$\begin{aligned} T:(x_{1}, x_{2}, x_{3},\ldots )\rightarrow \left( 0, x_{1}^{2}, a_{2}x_{2}, a_{3}x_{3},\ldots \right) , (x_{1}, x_{2}, x_{3},\ldots )\in B \end{aligned}$$

where $\{a_{i}\}$ is a sequence in (0,1) such that ${\prod ^{\infty }\nolimits_{i=2}(a_{i})=\frac{1}{2}}$.

It was proved by Goebel and Kirk (1972) that

(i) $${\left\| Tx-Ty\right\| \le 2\left\| x-y\right\| };$$
(ii) $${\left\| T^{n}x-T^{n}y\right\| \le 2\prod ^{n}_{i=2}(a_{i})\left\| x-y\right\| \;\forall x, y\in B\; \mathrm{and}\; n\ge 2}$$.

Now if we let $k^{\frac{1}{2}}_{1}=2$ such that ${k_{n}^{\frac{1}{2}} =2\prod ^{n}_{i=2}(a_{i}),\; \mathrm{for}\; n\ge 2}$, then

$$\begin{aligned} {\underset{n\rightarrow \infty }{\lim }k_{n}=\underset{n\rightarrow \infty }{\lim }\left( 2\prod ^{n}_{i=2}a_{i}\right) =1.} \end{aligned}$$

Similarly, if we let $\mu _{n}=k_{n}-1$, $\;\forall n\ge 1$, $\phi (t)=t^{2},\; \forall t\ge 0, k\in [0,1)$ and $\xi _{n}$ be a non-negative real sequence such that $\xi _{n}\rightarrow 0$, then $\forall x,y\in B$, $n\ge 1$, we have

$$\begin{aligned} \left\| T^{n}x-T^{n}y\right\| ^{2}\le \left\| x-y\right\| ^{2}+k\left\| x-y- \left( T^{n}x-T^{n}y\right) \right\| +\mu _{n}\phi (\left\| x-y\right\| )+\xi _{n}. \end{aligned}$$

### *Remark 2*

Note that, every nonexpansive mapping is k-strict pseudocontraction, k-strict pseudocontraction is asymptotically strict pseudocontraction mapping, asymptotically strict pseudocontraction mapping is ($k,~ \{\mu _{n}\},~\{\xi _{n}\},~ \phi )$-total asymptotically strict pseudocontraction mapping.

Throughout this paper, we adopt the notations; *I* is the identity operator, *Fix*(*T*) is the fixed point set of *T*, VIP(C,F) is the solution set of variational inequality problem [see Eq.  (1)], “$\rightarrow$” and “$\rightharpoonup$” denote the strong and weak convergence respectively, and $\omega _{\omega }(x_{n})$ denote the set of the cluster point of $\{x_{n}\}$ in the weak topology i.e., $\{ \exists x_{n_{j}}$ of $\{x_{n}\}$ such that $x_{n_{j}} \rightharpoonup x\}$.

Let *C* be a nonempty closed convex subset of *H* and $F:C \rightarrow H$ be a map. The variational inequality problem with respect to *C* and *F* is defined as search for $x^{*}\in C,$ such that

1 $$\begin{aligned} \left\langle Fx^{*}, x-x^{*}\right\rangle \ge 0, \quad \forall x\in C. \end{aligned}$$

The problem of solving a variational inequality problem of the form (1) has been intensively studied by numerous authors due to its various applications in several physical problems such as; in operational research, economics, engineering design etc., see for example Jianghua (2008), Noor (2007), Kinderlehrer and Stampacchia (1980) and the references therein.

It was Yamada (2001) proposed a hybrid steepest decent method for solving variational inequality problem, which generate a sequence $\{x_{n}\}$ by the following iterative algorithm:

2 $$\begin{aligned} \left\{ \begin{array}{ll} x_{0}\in H~ \mathrm{\;is \;arbitrarily;} \\ x_{n+1}=Tx_{n}-\mu _{n}\lambda _{n}F(Tx_{n}), \quad \forall n\ge 0, \end{array} \right. \end{aligned}$$

where *T* is nonexpansive mapping, *F* is *L*-Lipschitzian and $\eta$-strongly monotone with ${L>0, \eta >0, 0<\mu <\frac{2\eta }{L^{2}}}$ and $\lambda _{n}\subseteq (0,1)$ satisfying the following conditions:

3 $$\begin{aligned} \left\{ \begin{array}{l} \mathrm{(i)\quad} {\mathop{\lim }\nolimits_{{n \to \infty}}\lambda _{n}=0}, {\sum {\lambda _{n}}=\infty };\\ \mathrm{(ii) \quad either} {\sum {|\lambda _{n+1}-\lambda _{n}|}<\infty \;\mathrm{or}\;\mathop{\lim }\nolimits_{{n \to \infty}}\frac{\lambda _{n+1}}{\lambda _{n}}=1}. \end{array} \right. \end{aligned}$$

They showed that, the sequence $\{x_{n}\}$ generated by algorithm (2) converged strongly to the unique solution of variational inequality problem

4 $$\begin{aligned} \left\langle Fx^{*},x-x^{*}\right\rangle \ge 0, \quad \forall x\in Fix(T). \end{aligned}$$

Besides, he also proposed cyclic algorithm whose generate a sequence $\{x_{n}\}$ by

5 $$\begin{aligned} x_{x+1}=T^{\lambda _{n}}x_{n}=\left( I-\mu _{n}\lambda _{n} F\right) T_{[n]}x_{n},\quad \forall n\ge 0, \end{aligned}$$

where $T_{[n]}=T_{n(mod\, N)}$, he also got strong convergence results.

Marino and Xu (2006) introduced another algorithm for solving variational inequality problem, which generate a sequence $\{x_{n}\}$ by

6 $$\begin{aligned} \left\{ \begin{array}{ll} x_{0}\in H~ \mathrm{\;is \;arbitrarily;} \\ x_{n+1}=\alpha _{n}\gamma f(x_{n})+\left( I-\alpha _{n}A\right) T{x_{n}},&{} \end{array} \right. \end{aligned}$$

where *f* is a contraction, *A* is strongly positive bounded linear operator, *T* is a nonexpansive, $\{\alpha _{n}\}$ is a sequence in (0, 1) satisfying the conditions in Eq. (3), then they showed that, the sequence $\{x_{n}\}$ generated by algorithm (6), converged strongly to a common fixed point $x^{*}$ of *T* which solve the variational inequality problem

7 $$\begin{aligned} \left\langle (\gamma f-A)x^{*},x-x^{*}\right\rangle \le 0, \quad \forall x\in Fix(T). \end{aligned}$$

Tain (2010) combined algorithm (2) and (5), and he considered the following general iterative algorithm, which generate a sequence $\{x_{n}\}$ by:

8 $$\begin{aligned} \left\{ \begin{array}{ll} x_{0}\in H~ \mathrm{\;is \;arbitrarily;} \\ x_{n+1}=\alpha _{n}\gamma f(x_{n})+(I-\mu \alpha _{n}F)T{x_{n}},&{} \end{array} \right. \end{aligned}$$

where *T* is a nonexpansive, *f* is a contraction, *F* is *k* -Lipschitzian and $\eta$- strongly monotone with $k>0$, $\eta >0$, $0<\mu < \frac{2\eta }{k^{2}}$ and $\{\alpha _{n}\}$ is a sequence in (0, 1) satisfying the conditions in Eq. (3), then the sequence $\{x_{n}\}$ generated by algorithm (8), converged to a common fixed point $x^{*}$ of *T* which solves the variational inequality

9 $$\begin{aligned} \langle (\gamma f-\mu F)x^{*},x-x^{*}\rangle \le 0, \quad \forall x\in Fix(T). \end{aligned}$$

Tian and Di (2011) designed synchronal and cyclic algorithm based on the general iterative algorithm proposed by Tain (2010) for finding the common fixed point $x^{*}$ of finite family of strict pseudocontraction mapping, which is the solution of the variational inequality problem

10 $$\begin{aligned} \langle (\gamma f-\mu G)x^{*}, x-x^{*}\rangle \le 0, \quad \forall x\in \bigcap ^{N}_{i=1} Fix(T_{i}), \end{aligned}$$

and they obtained the strong convergent results as shown below:

### **Theorem 3**

(Synchronal Algorithm). *LetHbe a real Hilbert space and*$$T_{i}:H\rightarrow H$$*be a*$$k_{i}-$$*strict pseudocontraction, for some*$$k_{i}\subset (0,1)$$, $$(i=1,2,3,\ldots ,N)$$*such that*$$\bigcap ^{N}_{i=1} Fix(T_{i})\ne \emptyset$$, *let f be a contraction with coefficient*$$\beta \in (0,1)$$*and*$$\lambda _{i}$$*be a positive constant such that*$${\sum ^{N}_{i=1}\lambda _{i}=1}$$. *Let*$$G:H\rightarrow H$$*be a*$$\eta$$*-strongly monotone andL-Lipschitzian operator with*$$L>0$$*and*$$\eta >0.$$*Assume that*$$0<\mu <\frac{2\eta }{L^{2}},$$$${0<\gamma <\mu (\eta -\frac{\mu L^{2}}{2})/\beta =\frac{\tau }{\beta }}$$. *Given the initial guess*$$x_{0}\in H$$*chosen arbitrarily and given sequences*$$\{\alpha _{n}\}$$*and*$$\{\beta _{n}\}$$*in* (0, 1) *satisfying the following conditions:*

11 $$\begin{aligned} \left\{ \begin{array}{l} \mathrm{(i)} \quad {\mathop{\lim }\nolimits_{{n \to \infty}}\alpha _{n}=0}, \quad \sum {\alpha _{n}}=\infty ;\\ \mathrm{(ii)} \quad {\sum {|\alpha _{n+1}-\alpha _{n}|}<\infty , \quad \sum {|\beta _{n+1}-\beta _{n}|}<\infty }; \\ \mathrm{(iii)} \quad {0\le \max _{i} k_{i}\le \beta _{n}<a<1,\quad \forall n\ge 0}. \end{array} \right. \end{aligned}$$

*Let*$\{x_{n}\}$*be the sequence defined by*

12 $$\begin{aligned} \left\{ \begin{array}{ll} T^{\beta _{n}}=\beta _{n}I +(1-\beta _{n})\sum \nolimits ^{N}_{i=1}\lambda _{i}T_{i}; \\ x_{n+1}=\alpha _{n}\gamma f(x_{n})+\left( I-\alpha _{n}\mu G\right) T^{\beta _{n}}{x_{n}}.&{} \end{array} \right. \end{aligned}$$

*Then*$\{x_{n}\}$*converged strongly to a common point of*$\{T_{i}\}^{N}_{i=1}$*which solves the variational inequality problem* (10).

### **Theorem 4**

(Cyclic Algorithm) *LetHbe a real Hilbert space and*$$T_{i}:H\rightarrow H$$*be a*$$k_{i}-$$*strict pseudo-contraction for some*$$k_{i}\in (0,1)$$$$(i=1,2,3,\ldots ,N)$$*such that*$$\bigcap ^{N}_{i=1} Fix(T_{i})\ne \emptyset$$*and let f be a contraction with coefficient*$$\beta \in (0,1)$$. *Let*$$G:H\rightarrow H$$*be a*$$\eta$$*-strongly monotone andL-Lipschitzian operator with*$$L>0$$*and*$$\eta >0.$$*Assume that*$${0<\gamma <\mu \left( \eta -\frac{\mu L^{2}}{2}\right) /\beta =\frac{\tau }{\beta }}$$. *Given the initial guess*$$x_{0}\in H$$*chosen arbitrarily and given sequences*$$\{\alpha _{n}\}$$*and*$$\{\beta _{n}\}$$*in* (0, 1) *satisfying the following conditions:*

13 $$\begin{aligned} \left\{ \begin{array}{l} \mathrm{(i)} \quad {\mathop{\lim }\nolimits_{{n \to \infty}}\alpha _{n}=0}, \quad \sum {\alpha _{n}}=\infty ;\\ \mathrm{(ii)} \quad {\sum {|\alpha _{n+1}-\alpha _{n}|}<\infty , \ \mathrm{~or~} \mathop{\lim }\nolimits_{{n \to \infty}}\frac{\alpha _{n}}{\alpha _{n+1}}=1}; \\ \mathrm{(iii)}\quad \beta _{[n]}\in [k,1), \quad \mathrm{where~}k=\max \{k_{i}:1\le i\le N\}, \end{array} \right. \end{aligned}$$

*let*$\{x_{n}\}$*be the sequence defined by*

14 $$\begin{aligned} \left\{ \begin{array}{ll} A_{[n]}=\beta _{[n]}I +(1-\beta _{[n]})T_{[n]}; \\ x_{n+1}=\alpha _{n}\gamma f(x_{n})+(I-\alpha _{n}\mu G)A_{[n+1]}x_{n},&{} \end{array}\right. \end{aligned}$$

*where*$T_{[n]}=T_{i}$, *with i=n(mod N),*$1\le i\le N$, *namely*$T_{[n]}$*is one of*$T_{1},T_{2},T_{3},\ldots ,T_{N}$*circularly. Then*$\{x_{n}\}$*converged strongly to a common point of*$\{T_{i}\}^{N}_{i=1}$*which solve the variational inequality problem* (10).

And also Auwalu et al. (2013) proved the following results in real Banach space which is the generalization of Tian and Di (2011).

### **Theorem 5**

(Synchronal Algorithm) *LetEbe a realq-uniformly smooth Banach space, andCbe a nonempty closed convex subset ofE*. *Let*$T_{i}:C\rightarrow C$*be a*$k_{i}-$*strict pseudocontractions for some*$k_{i}\in (0,1)$, $(i=1,2,3,\ldots ,N)$*such that*$\bigcap ^{N}_{i=1} Fix(T_{i})\ne \emptyset$. *Letfbe a contraction with coefficient*$\beta \in (0,1)$*and*$\{\lambda _{i}\}_{i=1}^{N}$*be a sequence of positive number such that*${\sum ^{N}_{i=1}\lambda _{i}=1}$. *Let*$G:C\rightarrow C$*be an*$\eta$*-strongly accretive andL-Lipschitzian operator with*$L>0$ and $\eta >0.$*Assume that*$0<\mu <(q\eta /d_{q}L^{q})^{1/q-1}$, ${0<\gamma <\mu (\eta -d_{q}\mu ^{q-1}L^{q}/q)/\beta =\frac{\tau }{\beta }}$. *Let*$\{\alpha _{n}\}$*and*$\{\beta _{n}\}$*be sequences in (0,1) satisfying the following conditions:*

15 $$\begin{aligned} \left\{ \begin{array}{l} \mathrm{(k_{1})} \quad {\mathop{\lim }\nolimits_{{n \to \infty}}\alpha _{n}=0}, \ \sum {\alpha _{n}}=\infty ; \\ \mathrm{(k_{2})} \quad {\sum {|\alpha _{n+1}-\alpha _{n}|}<\infty ,\ \;\sum {|\beta _{n+1}-\beta _{n}|}<\infty }; \\ \mathrm{(k_{3})} \quad {0<k\le \beta _{n}<a<1, \quad \forall n\ge 0}, \;\mathrm{where}~ k=\min \{k_{i}: 1\le i\le N\};\\ \mathrm{(k_{4})} \quad {\alpha _{n},\beta _{n}\in [\mu , 1)} , \mathrm{\; where~} \mu \in \Big [\max \{0, 1-\left( \frac{\lambda q}{d_{q}}\right) ^\frac{1}{q-1}\},1\Big ). \end{array} \right. \end{aligned}$$

*Let*$\{x_{n}\}$*be a sequence defined by algorithm* (12), *then*$\{x_{n}\}$*converged strongly to a common fixed point of*$\{T_{i}\}^{N}_{i=1}$*which solve the variational inequality problem* (10).

Motivated by these two results, in this paper, we modified the algorithms of Tian and Di (2011) to the class of total asymptotically strict pseudocontraction mapping to solve the fixed-point problem as well variational inequality problem, this will be done in the frame work of real Hilbert space. By imposing some conditions, we obtained new strong convergence results. The results presented in this paper, not only extend and improve the results of Tian and Di (2011) but also extend, improve and generalize the results of; Yamada (2001), Marino and Xu (2006), Tain (2010) and Mainge (2009).

## Preliminaries

In the sequel we shall make use of the following lemmas in proving our main results.

### **Lemma 6**

( Marino and Xu 2007) *LetHbe a Hilbert space, there hold the following identities;*

(i) $$\left\| x-y\right\| ^{2}=\left\| x\right\| ^{2}-\left\| y\right\| ^{2}-2\left\langle x-y,y\right\rangle , \quad \forall x,y\in H;$$
(ii) $$\left\| tx+(1-t)y\right\| ^{2}=t\left\| x\right\| ^{2}+(1-t)\left\| y\right\| ^{2}-t(1-t)\left\| x-y\right\| ^{2}, \quad \forall t\in [0,1]\mathrm{~ and~} x,y\in H$$;
(iii) if $\{x_{n}\}$ is a sequence in *H* such that $x_{n}\rightharpoonup z,$ then
  $$\begin{aligned}\underset{n\rightarrow \infty }{\limsup }\left\| x_{n}-y\right\| ^{2}=\underset{n\rightarrow \infty }{\limsup }\left\| x_{n}-z\right\| ^{2}+\left\| z-y\right\| ^{2},\forall y\in H.\end{aligned}$$

### **Lemma 7**

(Chang et al. 2013) *LetCbe a nonempty closed convex subset of a real Hilbert spaceHand let*$T:C\rightarrow C$*be a* ($k,~ \{\mu _{n}\},~\{\xi _{n}\},~ \phi )$*-total asymptotically strict pseudocontraction mapping and uniformly L-Lipschitzian. Then*$I-T$*is demiclosed at zero in the sense that if*$\{x_{n}\}$*is a sequence inCsuch that*$x_{n}\rightharpoonup x^{*}$, *and*$\limsup\nolimits_{n\rightarrow \infty}\left\| (T^{n}-I)x_{n}\right\| =0$, *then*$(T-I)x^{*}=0.$

### **Lemma 8**

(Xu 2002) *Assume that*$\{a_{n}\}$*is a sequence of nonnegative real number such that*

$$\begin{aligned} a_{n+1}\le (1-\gamma _{n})a_{n}+\sigma _{n}, n\ge 0, \end{aligned}$$

*where*$\gamma _{n}$*is a sequence in* (0, 1) *and*$\sigma _{n}$*is a sequence of real number such that;*

(i) $$\mathop{\lim }\nolimits_{{n \to \infty}}\gamma _{n}=0\; \mathrm{and}\; \sum \gamma _{n}=\infty$$;
(ii) $\mathop{\lim }\nolimits_{{n \to \infty}}\frac{\sigma _{n}}{\gamma _{n}}\le 0$ or $\sum |\sigma _{n}|<\infty .$ Then $\mathop{\lim }\nolimits_{{n \to \infty}}a_{n}=0.$

### **Lemma 9**

(Tian and Di 2011) *Let*$F:H\rightarrow H$*be a*$\eta$*-strongly monotone andL-Lipschitzian operator with*$L>0$*and*$\eta >0$. *Assume that*${0<\mu <\frac{2\eta }{L^{2}}}$, ${\tau =\mu \left( \eta -\frac{2L^{2}\mu }{2}\right) }$*and*$0<t<1$. *Then*

$$\begin{aligned}\left\| (I-\mu tF)x-(I-\mu tF)y\right\| \le (I-\tau t)\left\| x-y\right\| .\end{aligned}$$

### **Lemma 10**

*Let*$S:C\rightarrow H$*be a uniformlyL-Lipschitzian mapping with*$L\in (0,1]$. *Define*$T:C\rightarrow H$*by*$T^{\beta _{n}}x=\beta _{n} x + (1-\beta _{n} )S^{n}x$*with*$\beta _{n}\in (0,1)$*and*$\forall x\in C$. *Then*$T^{\beta _{n}}$*is nonexpansive and*$Fix(T^{\beta _{n}})= Fix(S^{n})$.

### *Proof*

Let $x,y\in C,$ from Lemma [6(ii)], we have

$$\begin{aligned} \left\| T^{\beta _{n}}x-T^{\beta _{n}}y\right\| ^{2}&=\left\| \beta _{n}(x-y)+ (1-\beta _{n})(S^{n}x-S^{n}y)\right\| ^{2} \\&=\beta _{n}\left\| x -y \right\| ^{2}+(1-\beta _{n})\left\| S^{n}x-S^{n}y\right\| ^{2} \\&\quad -\,\beta _{n}(1-\beta _{n})\left\| (x-y)- \left( S^{n}x-S^{n}y\right) \right\| ^{2} \\&\le \beta _{n}\left\| x -y \right\| ^{2}+(1-\beta _{n})\left\| S^{n}x-S^{n}y\right\| ^{2} \\&\le \left( \beta _{n}+\left( 1-\beta _{n}\right) L^{2}\right) \left\| x-y\right\| ^{2}, \end{aligned}$$

since $$L\in (0,1]$$ and $$\beta _n \in (0,1)$$, it follow that, $$T^{\beta _{n}}$$ is nonexpansive, and it is not difficult to see that $$Fix(T^{\beta _{n}})= Fix(S^{n}).$$$$\square$$

### **Lemma 11**

(Tain 2010) *LetHbe a real Hilbert space,*$f:H\rightarrow H$*be a contraction with coefficient*$0<\alpha <1$*and*$F:H\rightarrow H$*be aL-Lipschitzian continuous operator and*$\eta$*-strongly monotone operator with*$L>0$*and*$\eta >0$. *Then for*$0<\gamma <\frac{\mu \eta }{\alpha }$,

$$\begin{aligned}\left\langle x-y, (\mu F-\gamma f)x-(\mu F-\gamma f)y\right\rangle \ge (\mu \eta -\gamma \alpha )\left\| x-y\right\| ^{2}.\end{aligned}$$

## Main results

In this section, we prove the following theorem which is the extension of the theorems (3) and (4).

### **Theorem 12**

*Let*$T:H\rightarrow H$*be a*$(k, \{\mu _{n}\}, \{\xi _{n}\}, \phi )$*-total asymptotically strict pseudocontraction mapping and uniformly M-Lipschitzian with*$\phi (t)=t^{2},\quad \forall t \ge 0$*and*$M\in (0,1]$. *Assume that*$Fix(T^{n})\ne \emptyset ,$*and letfbe a contraction with coefficient*$\beta \in (0,1)$, $G:H\rightarrow H$*be a*$\eta$*-strongly monotone andL-Lipschitzian operator with*$L>0$*and*$\eta >0$*respectively. Assume that*${0<\gamma <\mu (\eta -\frac{\mu L^{2}}{2})/\beta =\frac{\tau }{\beta }}$*and let*$x_{0}\in H$*be chosen arbitrarily,*$\{\alpha _{n}\}$*and*$\{\beta _{n}\}$*be two sequences in (0,1) satisfying the following conditions:*

16 $$\begin{aligned} \left\{ \begin{array}{l} \mathrm{(i)}\quad {\mathop{\lim }\nolimits_{{n \to \infty}}\alpha _{n}=0 \mathrm{~and~} \sum {\alpha _{n}}=\infty }; \\ \mathrm{(ii)} \quad{\sum {\left| \alpha _{n+1}-\alpha _{n}\right| }<\infty , \sum {\left| \beta _{n+1}-\beta _{n}\right| }<\infty }~\mathrm{and}~\sum {\left| 1-\beta _{n}\right| }<\infty ; \\ \mathrm{(iii)} \quad 0\le k\le \beta _{n}<a<1, \forall n\ge 0. \end{array} \right. \end{aligned}$$

*Let*$\{x_{n}\}$*be a sequence defined by*

17 $$\begin{aligned} \left\{ \begin{array}{ll} T^{\beta _{n}}=\beta _{n}I +(1-\beta _{n})T^{n}; \\ x_{n+1}=\alpha _{n}\gamma f(x_{n})+\left( I-\alpha _{n}\mu G \right) T^{\beta _{n}}{x_{n}},&{} \end{array} \right. \end{aligned}$$

*then*$\{x_{n}\}$*converges strongly to a common fixed of*$T^{n}$*which solve the variational inequality problem*

18 $$\begin{aligned} \langle (\gamma f-\mu G)x^{*}, x-x^{*}\rangle \le 0, \quad \forall x\in Fix\left( T^{n}\right) . \end{aligned}$$

### *Proof*

The proof is divided into five steps as follows.

**Step 1**. In this step, we show that

19 $$\begin{aligned} T^{\beta _{n}}\; \mathrm{is~nonexpansive~and}~ Fix(T^{\beta _{n}})=Fix\left( T^{n}\right) . \end{aligned}$$

The proof follows directly from Lemma (10).

**Step 2**. In this step, we show that

20 $$\begin{aligned} \{x_{n}\},\{T^{n}x_{n}\},\{f(x_{n})\}~\mathrm{and}~ \{GT^{n}x_{n}\} ~\mathrm{~are~ all~ bounded}. \end{aligned}$$

Let $x^{*}\in Fix(T^{n}),$ from (17) and Lemma (9), and the fact that *f* is a contraction, we have

$$\begin{aligned} \left\| x_{n+1}-x^{*}\right\|&= \left\| \alpha _{n}\gamma f(x_{n})+(I-\alpha _{n}\mu G)T^{\beta _{n}}{x_{n}}-x^{*}\right\| \\&=\left\| \alpha _{n}(\gamma f(x_{n})-\mu Gx^{*})+(I-\alpha _{n}\mu G)T^{\beta _{n}}{x_{n}}-(I-\alpha _{n}\mu G)x^{*}\right\| \\&\le (1-\alpha _{n}\tau )\left\| x_{n}-x^{*}\right\| +\alpha _{n}\left\| \gamma ( f(x_{n})-f(x^{*}))+\gamma f(x^{*})-\mu Gx^{*})\right\| \\&\le \left( 1-\alpha _{n}(\tau -\gamma \beta )\right) \left\| x_{n}-x^{*}\right\| +\alpha _{n}\left\| \gamma f(x^{*})-\mu Gx^{*})\right\| \\&\le \max \left\{ \left\| x_{n}-x^{*}\right\| ,\frac{\left\| \gamma f(x^{*})-\mu Gx^{*})\right\| }{(\tau -\gamma \beta )}\right\} . \end{aligned}$$

By using induction, we have

21 $$\begin{aligned} \left\| x_{n+1}-x^{*}\right\| \le \max \left\{ \left\| x_{0}-x^{*}\right\| ,\frac{\left\| \gamma f(x^{*})-\mu Gx^{*})\right\| }{(\tau -\gamma \beta )}\right\} . \end{aligned}$$

Hence $\{x_{n}\}$ is bounded, and also

22 $$\begin{aligned} \left\| T^{n}x_{n}-x^{*}\right\| ^{2}&\le \left\| x_{n}-x^{*}\right\| ^{2} + k\left\| x_{n}-x^{*}-\left( T^{n}x_{n}-x^{*}\right) \right\| ^{2}+\mu _{n}\phi \left( \left\| x_{n}-x^{*}\right\| \right) +\xi _{n}\nonumber \\&=\left\| x_{n}-x^{*}\right\| ^{2} + k\left\| x_{n}-x^{*}\right\| ^{2}+k\left\| T^{n}x_{n}-x^{*}\right\| ^{2}\nonumber \\&\quad +\,2k\left\| x_{n}-x^{*}\right\| \left\| T^{n}x_{n}-x^{*}\right\| +\mu _{n} \left\| x_{n}-x^{*}\right\| ^{2}+\xi _{n}\nonumber \\&\le (1+k+\mu _{n})\left\| x_{n}-x^{*}\right\| ^{2}+2k\left\| x_{n}-x^{*}\right\| \left\| T^{n}x_{n}-x^{*}\right\| \nonumber \\&\quad +\,k\left\| T^{n}x_{n}-x^{*}\right\| ^{2}+\xi _{n}. \end{aligned}$$

From (22), we deduce that

$$\begin{aligned}&(1-k)\left\| T^{n}x_{n}-x^{*}\right\| ^{2}-2k\left\| x_{n}-x^{*}\right\| \left\| T^{n}x_{n}-x^{*}\right\| \\&\quad -\,(1+k+\mu _{n})\left\| x_{n}-x^{*}\right\| ^{2}-\xi _{n}\le 0. \end{aligned}$$

This implies that

23 $$\begin{aligned} \left\| T^{n}x_{n}-x^{*}\right\|&\le \frac{k\left\| x_{n}-x^{*}\right\| }{(1-k)} \nonumber \\&\quad + \frac{\sqrt{4k^{2}\left\| x_{n}-x^{*}\right\| ^{2}+4(1-k)\{(1+k+\mu _{n})\left\| x_{n}-x^{*}\right\| ^{2}+\xi _{n}\}}}{2(1-k)} \nonumber \\&=\frac{k\left\| x_{n}-x^{*}\right\| + \sqrt{(1+(1-k)\mu _{n})\left\| x_{n}-x^{*}\right\| ^{2}+(1-k)\xi _{n}}}{(1-k)} \nonumber \\&\le \frac{k\left\| x_{n}-x^{*}\right\| + (1+(1-k)\mu _{n})\left\| x_{n}-x^{*}\right\| ^{2}+(1-k)\xi _{n}}{(1-k)} \left\| T^{n}x_{n}-x^{*}\right\| \le M^{*}, \end{aligned}$$

where $M^{*}$ is chosen arbitrarily such that

$$\begin{aligned}\sup \left( \frac{k\left\| x_{n}-x^{*}\right\| + (1+(1-k)\mu _{n}))\left\| x_{n}-x^{*}\right\| ^{2}+(1-k)\xi _{n}}{(1-k)}\right) \le M^{*}.\end{aligned}$$

It follows from (23) that $\{T^{n}x_{n}\}$ is bounded. Since *G* is *L*-Lipschitzian, *f* is contraction and the fact that $\{x_{n}\}, \{T^{n}x_{n}\}$ are bounded, it is easy to see that $\{GT^{n}x_{n}\}$ and $\{f(x_{n})\}$ are also bounded.

**Step 3.** In this step, we show that

24 $$\begin{aligned} \underset{n\rightarrow \infty }{\lim }\left\| x_{n+1}-x_{n}\right\| =0. \end{aligned}$$

Now,

$$\begin{aligned} x_{n+2}-x_{n+1}&=\Big (\alpha _{n+1}\gamma f(x_{n+1})+(I-\alpha _{n+1}\mu G)T^{\beta _{n+1}}{x_{n+1}}\Big ) \\&\quad -\,\Big (\alpha _{n}\gamma f(x_{n})+(I-\alpha _{n}\mu G)T^{\beta _{n}}{x_{n}}\Big ) \\&=\alpha _{n+1}\gamma ( f(x_{n+1})-f(x_{n}))+(\alpha _{n+1}-\alpha _{n})\gamma f(x_{n}) \\&\quad +\,(I-\alpha _{n+1}\mu G)T^{\beta _{n+1}}{x_{n+1}}-(I-\alpha _{n+1}\mu G)T^{\beta _{n}}{x_{n}} \\&\quad +\,(\alpha _{n}-\alpha _{n+1})\mu GT^{\beta _{n}}{x_{n}}, \end{aligned}$$

this turn to implies that

25 $$\begin{aligned} \left\| x_{n+2}-x_{n+1}\right\|\le & {}\, \alpha _{n+1}\gamma \beta \left\| x_{n+1}-x_{n}\right\| +(1-\alpha _{n+1}\tau )\left\| T^{\beta _{n+1}}{x_{n+1}}-T^{\beta _{n}}{x_{n}}\right\| \nonumber \\&+\, \left| \alpha _{n+1}-\alpha _{n}\right| \Big (\gamma \left\| f(x_{n})\right\| +\mu \left\| GT^{\beta _{n}}x_{n}\right\| \Big ) \nonumber \\\le & {} \,\alpha _{n+1}\gamma \beta \left\| x_{n+1}-x_{n}\right\| +(1-\alpha _{n+1}\tau )\left\| T^{\beta _{n+1}}{x_{n+1}}-T^{\beta _{n}}{x_{n}}\right\| \nonumber \\&+\, \left| \alpha _{n+1}-\alpha _{n}\right| N_{1}, \end{aligned}$$

where $N_{1}$ is chosen arbitrarily so that $\underset{n\ge 1}{\sup }\Big (\gamma \left\| f(x_{n})\right\| +\mu \left\| GT^{\beta _{n}}x_{n}\right\| \Big )\le N_{1}$.

On the other hand,

26 $$\begin{aligned} \left\| T^{\beta _{n+1}}x_{n+1}-T^{\beta _{n}}x_{n}\right\|&\le \left\| T^{\beta _{n+1}}{x_{n+1}}-T^{\beta _{n+1}}{x_{n}}\right\| +\left\| T^{\beta _{n+1}}{x_{n}}-T^{\beta _{n}}{x_{n}}\right\| \nonumber \\&\le \left\| x_{n+1}-x_{n}\right\| +\left| \beta _{n+1}-\beta _{n}\right| \left\| x_{n}\right\| +\left| 1-\beta _{n+1}\right| \left\| T^{n+1}x_{n}\right\| \nonumber \\&\quad + \,\left| (1-\beta _{n})\right| \left\| T^{n}x_{n}\right\| \nonumber \\&\le \left\| x_{n+1}-x_{n}\right\| +\left| \beta _{n+1}-\beta _{n}\right| N_{2}+\left| 1-\beta _{n+1}\right| N_{3} +\left| 1-\beta _{n}\right| N_{4}, \end{aligned}$$

where $N_{2,3,4}$ satisfy the following relations:

$$\begin{aligned}N_{2}\ge \underset{n\ge 1}{\sup }\left\| x_{n}\right\| ,\ N_{3}\ge \underset{n\ge 1}{\sup }\left\| T^{n+1}x_{n}\right\| \ \mathrm{and} \ N_{4}\ge \underset{n\ge 1}{\sup }\left\| T^{n}x_{n}\right\| \end{aligned}$$

respectively.

Now substituting (26) into (25), yields

$$\begin{aligned} \left\| x_{n+2}-x_{n+1}\right\|&\le \alpha _{n+1}\gamma \beta \left\| x_{n+1}-x_{n}\right\| +(1-\alpha _{n+1}\tau )\Big (\left\| x_{n+1}-x_{n}\right\| \\&\quad +\,\left| \beta _{n+1}-\beta _{n}\right| N_{2}+\left| 1-\beta _{n+1}\right| N_{3} +\left| 1-\beta _{n}\right| N_{4}\Big ) \\&\quad + \,\left| \alpha _{n+1}-\alpha _{n}\right| N_{1} \\&=(1+\alpha _{n+1}(\gamma \beta -\tau ))\left\| x_{n+1}-x_{n}\right\| + \left| \alpha _{n+1}-\alpha _{n}\right| N_{1} \\&\quad +\,(1-\alpha _{n+1}\tau )\Big (\left| \beta _{n+1}-\beta _{n}\right| N_{2}+\left| 1-\beta _{n+1}\right| N_{3} +\left| 1-\beta _{n}\right| N_{4}\Big ) \\&\le (1-\alpha _{n+1}(\tau -\gamma \beta )\left\| x_{n+1}-x_{n}\right\| \\&\quad +\,(1-\alpha _{n+1}\tau )\Big (\left| \beta _{n+1}-\beta _{n}\right| +\left| 1-\beta _{n+1}\right| +\left| 1-\beta _{n}\right| + \left| \alpha _{n+1}-\alpha _{n}\right| \Big )N_{5}, \end{aligned}$$

where $N_{5}$ choosing appropriately such that $N_{5}\ge \max \{N_{1},N_{2},N_{3},N_{4}\}$.

By Lemma (8) and (ii), it follows that

$$\begin{aligned} \underset{n\rightarrow \infty }{\lim }\left\| x_{n+1}-x_{n}\right\| =0. \end{aligned}$$

From Eq. (17), we have,

$$\begin{aligned} \left\| x_{n+1}-T^{\beta _{n}}x_{n}\right\|&=\left\| \alpha _{n}\gamma f(x_{n})+(I-\alpha _{n}\mu G)T^{\beta _{n}}x_{n}-T^{\beta _{n}}x_{n}\right\| \\&\le \alpha _{n}\left\| \gamma f(x_{n})-\mu GT^{\beta _{n}}x_{n}\right\| \rightarrow 0. \end{aligned}$$

On the other hand,

$$\begin{aligned} \left\| x_{n+1}-T^{\beta _{n}}x_{n}\right\|&=\left\| x_{n+1}-(\beta _{n}+(1-\beta _{n})T^{n})x_{n}\right\| \\&=\left\| (x_{n+1}-x_{n})+(1-\beta _{n})(x_{n}-T^{n}x_{n})\right\| \\&\ge (1-\beta _{n})\left\| x_{n}-T^{n}x_{n}\right\| -\left\| x_{n+1}-x_{n}\right\| , \end{aligned}$$

this implies that

$$\begin{aligned} \left\| x_{n}-T^{n}x_{n}\right\|&\le \frac{\left\| x_{n+1}-T^{\beta _{n}}x_{n}\right\| +\left\| x_{n+1}-x_{n}\right\| }{(1-\beta _{n})} \\&\le \frac{\left\| x_{n+1}-T^{\beta _{n}}x_{n}\right\| +\left\| x_{n+1}-x_{n}\right\| }{(1-a)}\rightarrow 0. \end{aligned}$$

From the boundedness of $\{x_{n}\}$, we deduce that $\{x_{n}\}$ converges weakly. Now assume that $x_{n}\rightharpoonup p$, by Lemma (7) and the fact that $\left\| x_{n}-T^{n}x_{n}\right\| \rightarrow 0$, we obtain $p\in Fix\left( T^{n}\right)$. So, we have

27 $$\begin{aligned} \omega _{\omega }(x_{n})\subset Fix\left( T^{n}\right) . \end{aligned}$$

By Lemma (11) it follows that $(\gamma f-\mu G)$ is strongly monotone, so the variational inequality (18) has a unique solution $x^{*}\in Fix(T^{n})$.

**Step 4**. In this step, we show that

28 $$\begin{aligned} \underset{n\rightarrow \infty }{\limsup }\left\langle (\gamma f-\mu G)x^{*}, x_{n}-x^{*}\right\rangle \le 0. \end{aligned}$$

The fact that $\{x_{n}\}$ is bounded, we have $\{x_{n_{i}}\}\subset \{x_{n}\}$ such that

$$\begin{aligned}\underset{n\rightarrow \infty }{\limsup }\left\langle (\gamma f-\mu G)x^{*}, x_{n}-x^{*}\right\rangle =\underset{i\rightarrow \infty }{\limsup }\left\langle (\gamma f-\mu G)x^{*}, x_{n_{i}}-x^{*}\right\rangle \le 0.\end{aligned}$$

Suppose without loss of generality that $x_{n_{i}}\rightharpoonup x$, from (27), it follows that $x\in Fix (T^{n})$. Since $x^{*}$ is the unique solution of (17), implies that

$$\begin{aligned} \underset{n\rightarrow \infty }{\limsup }\left\langle (\gamma f-\mu G)x^{*}, x_{n}-x^{*}\right\rangle&=\underset{i\rightarrow \infty }{\limsup }\left\langle (\gamma f-\mu G)x^{*}, x_{n_{i}}-x^{*}\right\rangle . \\&=\left\langle (\gamma f-\mu G)x^{*}, x-x^{*}\right\rangle \le 0. \end{aligned}$$

**Step 5.** In this step, we show that

29 $$\begin{aligned} \underset{n\rightarrow \infty }{\lim }\left\| x_{n}-x^{*}\right\| =0. \end{aligned}$$

By Lemma (9) and the fact that *f* is a contraction, we have

$$\begin{aligned} \left\| x_{n+1}-x^{*}\right\| ^{2}&=\left\| \alpha _{n}(\gamma f(x_{n})-\mu Gx^{*})+(I-\alpha _{n}\mu G)T^{\beta _{n}}x_{n}-(I-\alpha _{n}\mu G)x^{*}\right\| ^{2} \\&\le \left\| (I-\alpha _{n}\mu G)T^{\beta _{n}}x_{n}-(I-\alpha _{n}\mu G)x^{*}\right\| ^{2} \\&\quad +\,2\alpha _{n}\left\langle \gamma f(x_{n})-\mu Gx^{*},x_{n+1}-x^{*}\right\rangle \\&\le (1-\alpha _{n}\tau )^{2} \left\| x_{n}-x^{*}\right\| ^{2}+2\alpha _{n}\gamma \left\langle f(x_{n})-f(x^{*}),x_{n+1}-x^{*}\right\rangle \\&\quad +\,2\alpha _{n}\left\langle \gamma f(x^{*})-\mu Gx^{*},x_{n+1}-x^{*}\right\rangle \\&\le (1-\alpha _{n}\tau )^{2} \left\| x_{n}-x^{*}\right\| ^{2}+2\alpha _{n}\beta \gamma \left\| x_{n}-x^{*}\right\| \left\| x_{n+1}-x^{*}\right\| \\&\quad +\,2\alpha _{n}\left\langle \gamma f(x^{*})-\mu Gx^{*},x_{n+1}-x^{*}\right\rangle \\&\le (1-\alpha _{n}\tau )^{2} \left\| x_{n}-x^{*}\right\| ^{2}+\alpha _{n}\beta \gamma \Big ( \left\| x_{n}-x^{*}\right\| ^{2}+\left\| x_{n+1}-x^{*}\right\| ^{2}\Big ) \\&\quad +\,2\alpha _{n}\left\langle \gamma f(x^{*})-\mu Gx^{*},x_{n+1}-x^{*}\right\rangle , \end{aligned}$$

this implies that

$$\begin{aligned}\left\| x_{n+1}-x^{*}\right\| ^{2}&\le \frac{\Big ( (1-\alpha _{n}\tau )^{2} +\alpha _{n}\beta \gamma \Big )\left\| x_{n}-x^{*}\right\| ^{2}}{(1-\alpha _{n}\gamma \beta )} \\&\quad +\,\frac{2\alpha _{n}\left\langle \gamma f(x^{*})-\mu Gx^{*},x_{n+1}-x^{*}\right\rangle }{(1-\alpha _{n}\gamma \beta )} \\&\le \Big ( 1-(2\tau -\gamma \beta )\alpha _{n}\Big )\left\| x_{n}-x^{*}\right\| ^{2}+\frac{(\alpha _{n}\tau )^{2}}{(1-\alpha _{n}\gamma \beta )}\left\| x_{n}-x^{*}\right\| ^{2} \\&\quad +\,\frac{2\alpha _{n}\left\langle \gamma f(x^{*})-\mu Gx^{*},x_{n+1}-x^{*}\right\rangle }{(1-\alpha _{n}\gamma \beta )}, \end{aligned}$$

this implies that

$$\begin{aligned}\left\| x_{n+1}-x^{*}\right\| ^{2}\le (1-\gamma _{n})\left\| x_{n}-x^{*}\right\| ^{2}+\sigma _{n}, \end{aligned}$$

where

$$\begin{aligned} \gamma _{n}:= & {} \,(2\tau -\gamma \beta )\alpha _{n} \ \ \mathrm{and} \\ \sigma _{n}:= & {} \,\frac{\alpha _{n}}{(1-\alpha _{n}\gamma \beta )}\Big (\alpha _{n}\tau ^{2}\left\| x_{n}-x^{*}\right\| ^{2}+2\left\langle \gamma f(x^{*})-\mu Gx^{*}, x_{n+1}-x^{*}\right\rangle \Big ). \end{aligned}$$

From [12(i)], it follows that

$$\begin{aligned}&\mathop{\lim }\limits_{{n \to \infty}}\gamma _{n}= 0, \\&\sum \gamma _{n}=\infty ,\\&\frac{\sigma _{n}}{\gamma _{n}}=\frac{1}{(2\tau -\gamma \beta )(1-\alpha _{n}\gamma \beta )}\Big (\alpha _{n}\tau ^{2}\left\| x_{n}-x^{*}\right\| ^{2}+2\left\langle \gamma f(x^{*})-\mu Gx^{*},x_{n+1}-x^{*}\right\rangle \Big ). \end{aligned}$$

Thus $${\mathop{\lim }\nolimits_{{n \to \infty}}\frac{\sigma _{n}}{\gamma _{n}}\le 0}$$.

Hence by Lemma (8), it follows that $x_{n}\rightarrow x^{*}$ as $n\rightarrow \infty$. $\square$

### **Corollary 13**

*LetBbe a unit ball in a real Hilbert space*$l_{2}$, *and let the mapping*$T:B\rightarrow B$*be defined by*

$$\begin{aligned} T:\left( x_{1}, x_{2}, x_{3},\ldots \right) \rightarrow \left( 0, x_{1}^{2}, a_{2}x_{2}, a_{3}x_{3},\ldots \right) , \left( x_{1}, x_{2}, x_{3},\ldots \right) \in B, \end{aligned}$$

*where*$\{a_{i}\}$*is a sequence in* (0, 1) *such that*${\prod ^{\infty }_{i=2}(a_{i})=\frac{1}{2}}$. *Let,*$f, G,\gamma , \{\alpha _{n}\}, \{\beta _{n}\}$*be as in theorem *(12)*. Then the sequence*$\{x_{n}\}$*define by algorithm* (17), *converges strongly to a common fixed point of*$~T^{n}$*which solve the variational inequality problem *(18)*.*

### *Proof*

By example (1), it follows that *T* is $(k, \{\mu \}, \{\xi _{n}\}, \phi )$-total asymptotically strict pseudocontraction mapping and uniformly *M*-Lipschitzian with $M= {2\prod\nolimits ^{n}_{i=2}(a_{i})}$. Hence, the conclusion of this corollary, follows directly from theorem (12). $\square$

### **Corollary 14**

*LetHbe a real Hilbert space and*$$T:H\rightarrow H$$*be a*$$(k, \{k_{n}\})$$*- asymptotically strict pseudocontraction mapping and uniformlyM-Lipschitzian with*$$M\in (0,1]$$. *Assume that*$$Fix(T^{n})\ne \emptyset$$, *and Let*$$f, G, \gamma$$$$\{\alpha _{n}\}$$*and*$$\{\beta _{n}\}$$*be as in theorem *(12)*. Then, the sequence*$$\{x_{n}\}$$*generated by algorithm* (17), *converges strongly to a common fixed point of*$$~T^{n}$$*which solve the variational inequality problem* (18).

### **Corollary 15**

(Tain 2010) *Let the sequence*$\{x_{n}\}$*be generated by the mapping*

$$\begin{aligned} x_{n+1}=\alpha _{n} \gamma f(x_{n})+(I-\mu \alpha _{n}F)Tx_{n}, \end{aligned}$$

*whereTis nonexpansive,*$\alpha _{n}$*is a sequence in (0,1) satisfying the conditions in Eq.* (11). *It was proved in* Tain (2010) *that*$\{x_{n}\}$*converged strongly to the common fixed point*$x^{*}$*ofT*, *which is the solution of variational inequality problem*

30 $$\begin{aligned} \langle (\gamma f- \mu F)x^{*},x-x^{*}\rangle \le 0, \quad \forall x\in Fix(T). \end{aligned}$$

### *Proof*

Take n=1, $k=\mu _{n}=\xi _{n}=0$ and $F=G$ in theorem (12). Therefore all the conditions in theorem (12) are satisfied. Hence the conclusion of this corollary follows directly from theorem (12). $\square$

### **Corollary 16**

(Marino and Xu 2006) *Let the sequence*$\{x_{n}\}$*be generated by*

$$\begin{aligned}x_{n+1}=\alpha _{n} \gamma f(x_{n})+(I-\alpha _{n}A)Tx_{n},\end{aligned}$$

*whereTis nonexpansive and the sequence*$\alpha _{n}\subset (0,1)$*satisfy the conditions in Eq.* (16). *Then it was proved in* Marino and Xu (2006) *that*$\{x_{n}\}$*converged strongly to*$x^{*}$*which solve the variational inequality*

31 $$\begin{aligned} \langle (\gamma f- A)x^{*},x-x^{*}\rangle \le 0, \quad \forall x\in Fix(T). \end{aligned}$$

### *Proof*

Take n=1, $\mu _{n}=\xi _{n}=0$ and $\mu =1$ and $G=A$ in theorem (12). Therefore all the conditions in theorem (12) are satisfied. Hence the conclusion of this corollary follows directly from theorem (12). $\square$

### **Corollary 17**

(Yamada 2001) *Let the sequence*$\{x_{n}\}$*be generated by*

$$\begin{aligned}x_{n+1}=Tx_{n}-\mu \lambda _{n}F(Tx_{n}),\end{aligned}$$

*whereTis nonexpansive mapping onH*, *Fis L-Lipschitzian and*$\eta$*-strongly monotone with*$L>0, \eta >0$*and*$0<\mu <\frac{2\eta }{L^{2}}$, *if the sequence*$\lambda _{n}\subset (0,1)$*satisfies the conditions in* (3). *Then, it was proved by* Yamada (2001) *that*$\{x_{n}\}$*converged strongly to the unique solution of the variational inequality*

32 $$\begin{aligned} \langle Fx^{*},x-x^{*}\rangle \ge 0,\quad \forall x\in Fix(T). \end{aligned}$$

### *Proof*

Take $n=1$, $k=\mu _{n}=\xi _{n}=0$ and also take $\gamma =0$, $\beta _{n}=0$ and $G=F$. Therefore all the conditions in theorem (12) are satisfied. Hence the result follows directly from theorem (12). $\square$

## Conclusion

In this paper, we modified the algorithms by Tian and Di (2011) in order to include the class of total asymptotically strict pseudocontraction mapping to solve the fixed-point problem as well variational inequality problem, this was done in the frame work of real Hilbert spaces. By imposing some conditions, we also obtained some new strong convergence results. Further we state that the results which were presented in this paper, not only extend and improve the results (Tian and Di 2011) but also extend, improve and generalize the results of; Yamada (2001), Marino and Xu (2006), Tain (2010) and Mainge (2009).
